# Ag/MnO_2_ Composite Sheath-Core Structured Yarn Supercapacitors

**DOI:** 10.1038/s41598-018-31611-2

**Published:** 2018-09-06

**Authors:** Ji Hwan Kim, Changsoon Choi, Jae Myeong Lee, Mônica Jung de Andrade, Ray H. Baughman, Seon Jeong Kim

**Affiliations:** 1 Center for Self-powered Actuation, Department of Biomedical Engineering, Hanyang University, Seoul, 04763 Korea; 2 Division of Smart Textile Convergence Research, Daegu Gyeongbuk Institute of Science and Technology (DGIST), Daegu, 42988 Korea; 3 The Alan G. MacDiarmid NanoTech Institute, University of Texas at Dallas, Richardson, TX 75083 USA

## Abstract

One-dimensional (1D) yarn or fiber-based supercapacitors that have small diameter, volume and high mechanical strength are needed due to the demands on power source for wearable electronics, micro-devices, and implantable medical devices. The composite sheath is fabricated on a commercially available CNT yarn substrate by alternating depositions of MnO_2_ and Ag layers. Synergistic effect of high loading level of pseudocapacitive MnO_2_ and reasonably improved rate-capability are achieved. In the composite sheath, the interconnected networks provide electrical contact between MnO_2_ aggregates and adjacent Ag layer. The conductive Ag inter layers shorten the solid-state charge diffusion length in the MnO_2_. Moreover, generated electrons during the charge/discharge process can be collected rapidly by the adjacent Ag layer, therefore, the great extents of MnO_2_ could be loaded onto the surface of CNT core fiber electrode without a significant rate-capability degradation. Due to the high MnO_2_ loading level, the composite sheath-core yarn supercapacitor showed excellent specific areal capacitance (322.2 mF/cm^2^) and according energy density (18.3 µWh/cm^2^).

## Introduction

One-dimensional (1D) yarn or fiber shaped micro-supercapacitors that possess small diameter, volume and high mechanical degree of freedom, like flexibility or stretchability are highly needed due to the sudden high demand on appropriate power source for wearable electronics, micro-devices, and implantable medical devices^1–10^. In addition, various methods are being studied to improve energy storage performance^11–13^. However, actualizing practically available yarn supercapacitors still remain as challenge because of their limited charge storage capability. As one of the promising pseudocapacitive active materials, MnO_2_ has been extensively studied due to its high theoretical energy storage capability, low cost, and environmental friendliness. However, it has a poor electrical conductivity^14^, which significantly limits the solid-state charge transport and leads to a significant performance degradation when the MnO_2_ loading density increases^15^. Although a nanoscopically thin MnO_2_ film can reduce the solid-state ion diffusion lengths and provide high specific capacitance per MnO_2_ weight, because of extremely low loading mass, the overall energy and power densities (including all components) of MnO_2_-based devices have been low^16^. This is an important issue because the performances normalized by the not active material alone but the dimension of the entire device, including other cell components like current collector, electrolyte, and separator, can give a practical picture of an actual device application^17^. Unfortunately, especially for sheath (pseudocapacitive active materials) – core (current collecting conductive yarn) structured yarn supercapacitors, the mentioned high MnO_2_ loading issue becomes more critical. Due to the limited surface area and volume of 1D yarn electrodes with micro-diameters, only the surface area of the core yarn is used as active material loading site while the core part does not participate in energy storage reaction^1^. For storage performance resulting from high active material loading, thick and bulk pseudocapacitive sheath formation is needed onto the current collector. However, it leads supercapacitors’ degraded rate-capability or specific power, and inhibition of solid-state charge diffusion process^14^. In addition, the specific surface area and according ion accessibility of the active material are also limited by thick sheath.

From this point, we introduce a novel composite sheath structured yarn supercapacitor, which consists of electrochemically active MnO_2_ and electrically conductive Ag. The composite sheath is fabricated on commercially available CNT yarn substrates by alternating the electrochemical deposition of MnO_2_ and Ag layers. In synergistic combination of high loading level of pseudocapacitive MnO_2_ and reasonably improved rate-capability are achieved. In the composite sheath, the interconnected composite network provides electrical contact between MnO_2_ aggregates and adjacent Ag layer. The Ag conductive inter layers shorten the solid-state charge diffusion length in the MnO_2_. Moreover, generated electrons during the discharging process can be effectively collected by the adjacent Ag layer, therefore, the great extents of MnO_2_ (about 71.5 wt%) could be loaded onto the surface of CNT core yarn electrode without a significant rate-capability degradation or an impediment of mechanical flexibility. Thanks to high active material loading level, the composite sheath-core yarn supercapacitor showed excellent charge storage capabilities (C_A_ = 322.3 mF cm^−2^, C_V_ = 208.1 F cm^−3^) and according energy densities (E_A_ = 18.3 µWh cm^−2^, E_V_ = 11.8 mWh cm^−3^. More importantly, the composite sheath supercapacitor exhibited enhanced rate-capability, which is about 40% of the initial capacitance, and was retained when scan rate increased from 10 to 100 mV s^−1^, which is two-fold higher than pristine MnO_2_ sheath-core structured supercapacitor.

## Results and Discussion

Previously reported literatures about yarn or fiber supercapacitors are mostly based on core-sheath structure configuration, where only the surface area of the electrically conductive core yarn is used as a loading site, increasing the MnO_2_ loading level leads to a thick film formation with poor electrical conductivity. Unfortunately, from a view point of electrochemical reaction, this thick active material coating has several disadvantages such as longer solid-state ion diffusion length, high resistance, and low electrochemically active surface area. For this reason, previous reported plain core-sheath structured yarn supercapacitors which adopted the MnO_2_ as an active material suffered from low MnO_2_ loading levels mostly less than 20 wt%^2,18,19^. The high active material loading without impairing the electrochemical performance or the mechanical strength is a very important research issue. Although a recent research achieved a record high MnO_2_ loading level (~93 wt%) inside the CNT yarn electrode by introducing liquid-state biscrolling technology, the limitation of using expensive, non-mass productive CNT sheets that are drown from CNT forest still exists^20–22^.

**Figure 1:**
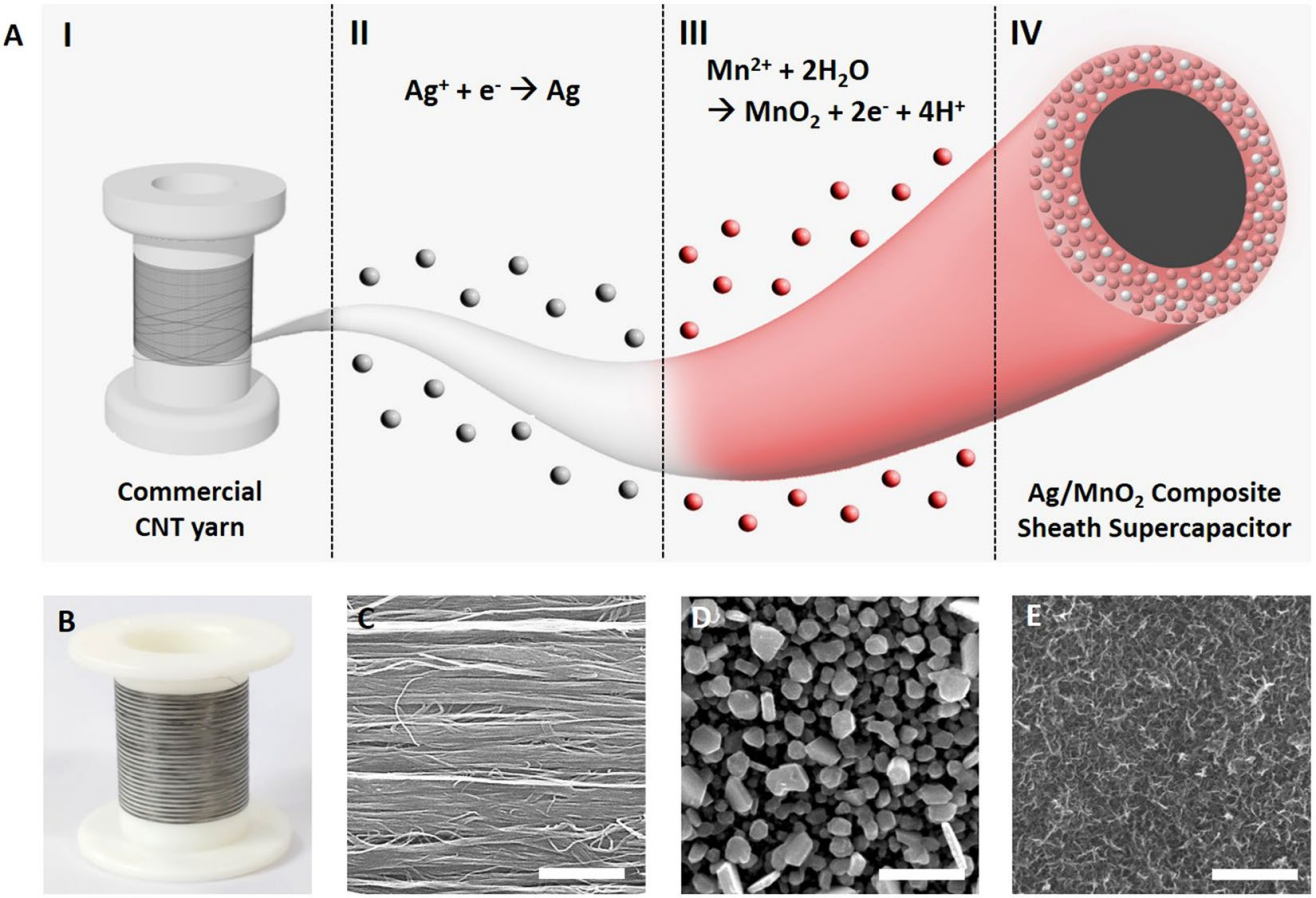
(**A**) Schematic illustration showing Ag/MnO_2_ composite sheath yarn fabrication process. Alternative and repetitive deposition is performed by applying square wave potential to deposit the MnO_2_ and Ag composite sheath onto the surface of commercial CNT yarn. (**B**) The optical image of 10 meters long commercial CNT yarn. The magnified surface SEM images showing (**C**) pristine CNT yarn, (**D**) Ag deposition, and (**E**) MnO_2_ deposition. (Scale bar = 500 nm).

To address the limitations stated above, we propose a structural approach on yarn coating. The fabrication of the Ag/MnO_2_ composite sheath on top of the commercially available CNT yarns aimed to maximize the pseudocapacitive active material (MnO_2_) loading and maintain electrical conductivity, simultaneously. The pseudocapacitive MnO_2_ and electrically conductive Ag were alternately deposited by electrochemical deposition method as shown in scheme Fig. 1A. We started from tens of meter-long, mechanically strong, and commercially available CNT yarns which required no further pre-treatment (Fig. 1B). Highly aligned CNT bundles characterized by SEM image shown in Fig. 1C can provide effective electron pathway and mechanical strength in yarn length direction^23^. During the depositions, square wave potential was applied for core CNT yarn electrodes to form alternate MnO_2_ and Ag based composite sheath. The magnified surface images of the Ag and MnO_2_ deposites were shown in Fig. 1D,E, respectively. Rough surface Ag film was formed (Fig. 1D) while highly porous, even, and flower-like MnO_2_ was observed (Fig. 1E), which is a morphological characteristic of the MnO_2_ deposite^24^. Deposited amount of the sheath shell was controlled by deposition time or deposition number. As the deposition time for MnO_2_ increases, the specific capacitance also increases while the rate-capability decreases, which is obtained by low-rate CV area (at 10 mV s^−1^) as shown in Fig. S1A. Reversely, increasing Ag deposition number results in capacitance decrease and retention increase (Fig. S1B). For further electrochemical characterization, optimized deposition for composite sheath was applied (total of MnO_2_ deposition time for 50 minutes, Ag deposition time 90 seconds.) Deposition of composite sheath was followed by repetitive deposition method that does not require any post treatment (vacuum or annealing treatment).

**Figure 2:**
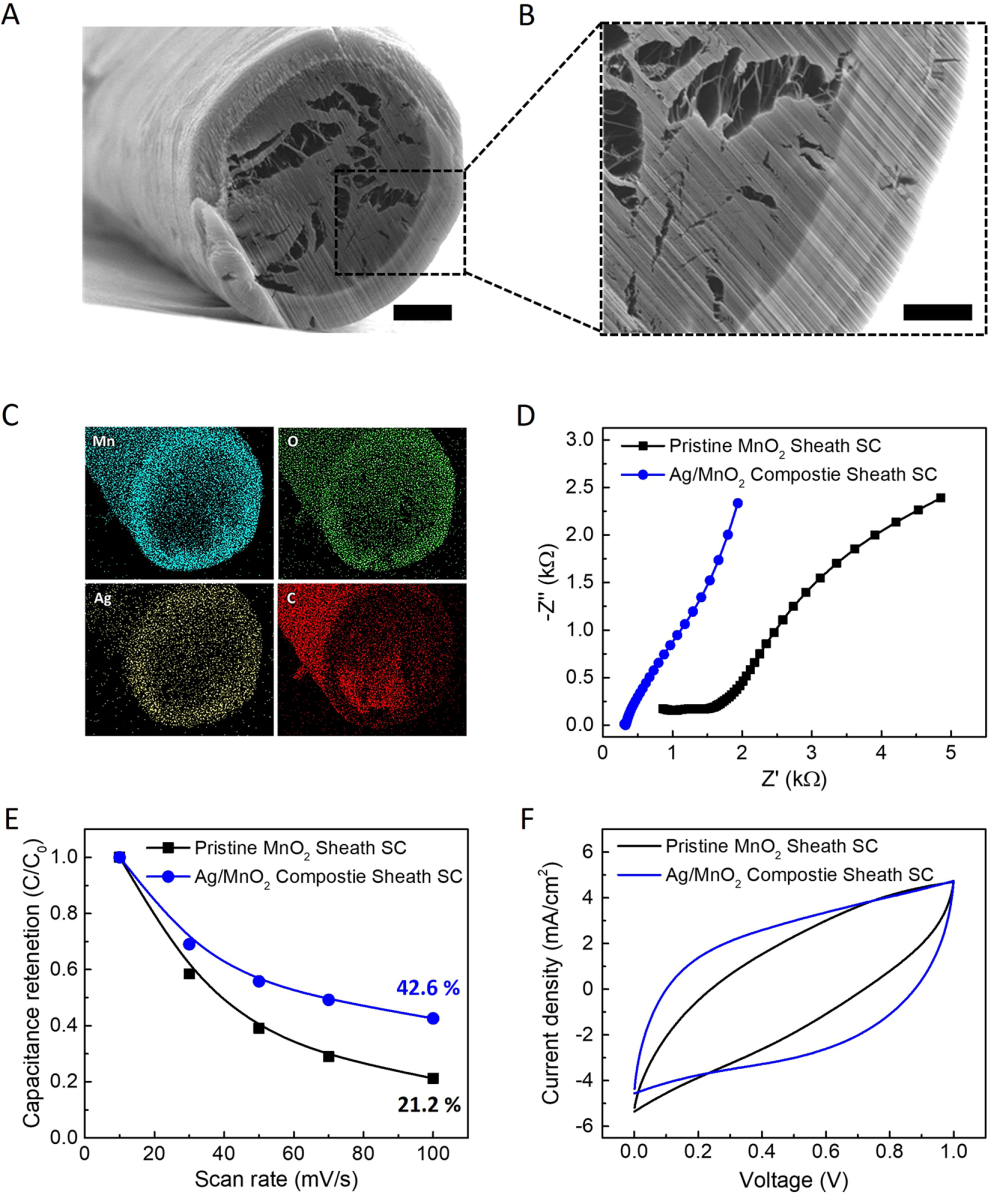
(**A**) Cross-sectional SEM image of Ag/MnO_2_ composite sheath yarn electrode. (Scale bar = 10 μm). (**B**) Magnified SEM image of Ag/MnO_2_ composite sheath yarn electrode’s core and sheath. (Scale bar = 4 μm). (**C**) Elemental mapping of Mn, O, Ag and C by EDS over the cross-section image. (**D**) Nyquist curves for the frequency range from 0.01 to 100 kHZ. The system comprises two symmetric Ag/MnO_2_ composite electrodes coated by PVA-LiCl gel electrolyte. (**E**) Calculated capacitance retention of the solid-state pristine MnO_2_ sheath and Ag/MnO_2_ composite sheath supercapacitor on various scan rate (measured from 10 to 100 mV s^−1^). (**F**) The comparison of CV curves (at 100 mV s^−1^) pristine MnO_2_ sheath (black line) and Ag/MnO_2_ composite sheath (blue line) supercapacitors. All measurements are performed in two electrode configuration yarns coated with gel based solid electrolyte.

Cross-sections of composite sheath-core yarns were prepared by cutting them along their diameters using a Ga ion beam (7 nA beam current) in a Focused Ion Beam (FIB, Nova 200) operated at 30 kV. Then, the cut areas were cleaned via Ga ion polishing by etching several micrometers of yarn length with consecutively decreasing ion beam-currents ranging from 5.0 to 0.3 nA. The cleaned-cut yarns were next transferred to a Zeiss Supra 40 SEM to perform the microscopy (at 15 kV) and elemental EDAX mapping analysis (at 20 kV). Clear views of the yarn’s cross-sections were attained by orienting the yarn’s cut plane parallel to the electron-beam final aperture/detector in the SEM. Related SEM images and EDAX mapping results are provided in Figs 2A–C and S2. We define the sheath/core ratio as the ratio of the sheath thickness to the yarn diameter, as measured by using SEM images for twisted yarn. From SEM micrograph observation, it was estimated that the 50 μm composite yarn has a sheath/core ratio of about 0.106. EDX mapping and quantitative analyses (total of 3 areas per each sheath and core regions) showed almost no infiltration of the MnO_2_ or Ag into the fiber core (Mn and Ag atomic concentration at sheath and core are 18.5(±2.7) at.% and 1.7(±0.3) at.% against <4 at.% and <0.5 at.%, respectively, as shown in Fig. S2). XRD pattern of hybrid MnO_2_/Pt supercapacitor is shown in Fig. S3. From the electrochemical impedance spectroscopy (EIS) measurement (Fig. 2D), normalized equilibrium series resistance (ESR) at 1 kHz for solid-state composite sheath-core supercapacitor is as small as 0.3 kΩ and it exhibited high slope of Nyquist curve, implying also good capacitive characteristic. On the other hand, the pristine MnO_2_ sheath-core yarn supercapacitor showed larger ESR value (1.3 kΩ at 1 kHz) and lower Nyquist slope, which originated from long solid-state ion diffusion length and limited ion accessibility by thick MnO_2_ coating. As a result, the composite sheath structured supercapacitor exhibited improved rate-capability that 42.6% capacitance was retained when the scan rate was increased from 10 to 100 mV s^−1^ while the pristine MnO_2_ retained only 21.2% not showing box-like CV curves (Figs 2E and S4). The CV curves for composite and pristine sheath structured supercapacitors compared in Fig. 2F also support the improved rate-capability performance.

**Figure 3:**
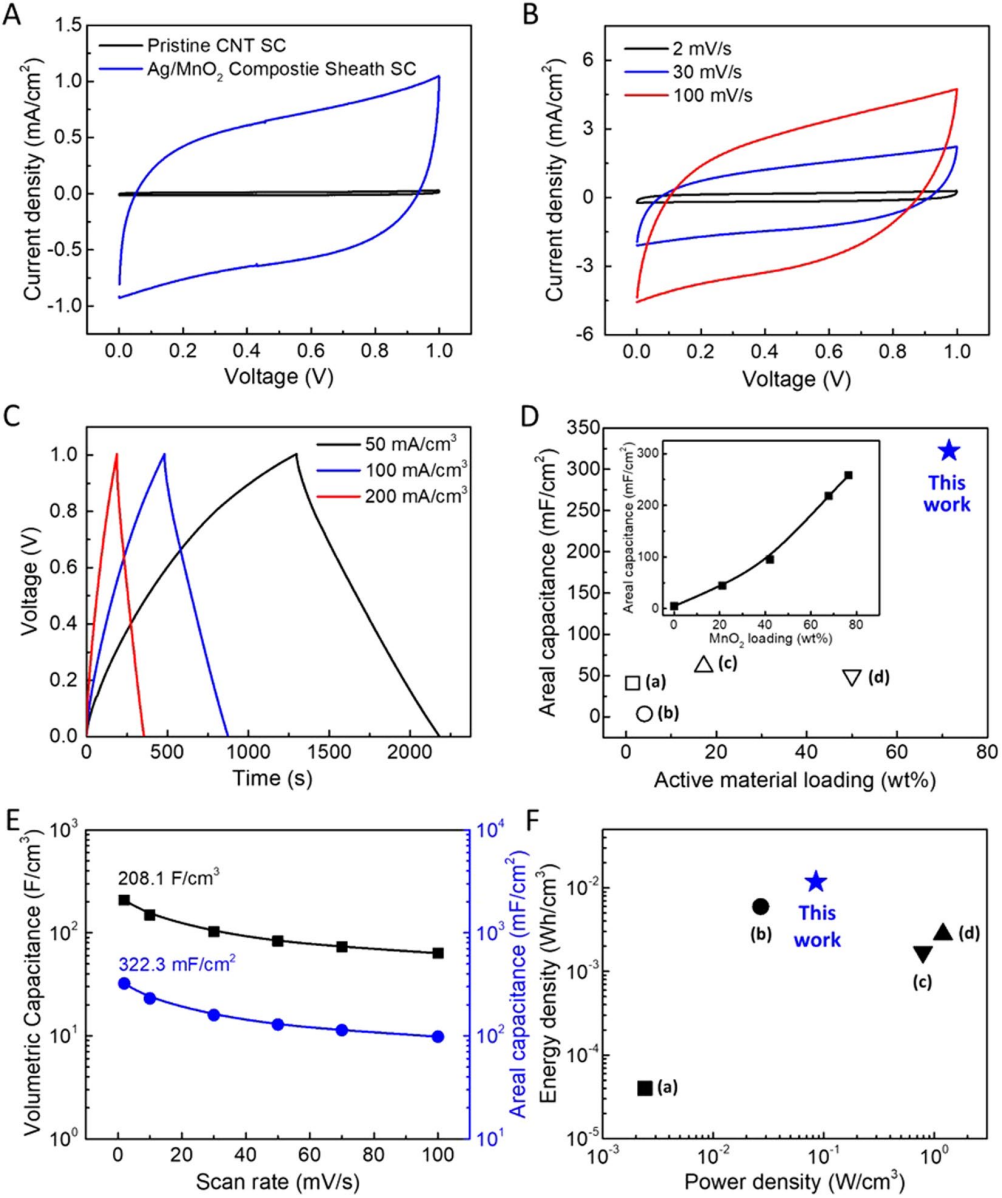
(**A**) CV curves of pristine CNT and Ag/MnO_2_ composite sheath supercapacitor measured at 10 mV s^−1^. (**B**) CV (measured from 2 to 100 mV s^−1^) and (**C**) Galvano-static curves (measured from 50 to 200 mA cm^−3^) of Ag/MnO_2_ composite sheath supercapacitor. The supercapacitors comprise two symmetric electrodes coated by PVA/LiCl gel electrolyte. (**D**) Areal capacitances of yarn supercapacitor versus active material loading of composite sheath yarn supercapacitor compared with reported yarn supercapacitors with various amounts of active materials loading: (a) 1.45 wt% MnO_2_ coated CNT/nylon coil fiber^5^, (b) 4.1 wt% MnO_2_ coated CNT yarn^1^, (c) 17.2 wt% MnO_2_ coated CNT coil yarn^4^, (d) 50 wt% polyaniline coated CNT/rubber fiber^6^. The inset shows areal capacitances with increasing MnO_2_ loading in pristine MnO_2_ sheath supercapacitor. (**E**) Areal and volumetric capacitances from 2 to 100 mV s^−1^. (**F**) Ragone plots comparing volumetric energy and power densities for the present all-solid-state supercapacitor: (a) 2.44 mW cm^−3^–0.04 mWh cm^−3^ ZnO-doped MnO_2_ Core/shell nanocables^8^, (b) 26.9 mW cm^−3^–5.97 mWh cm^−3^ poly(vinyl alcohol)/graphene hybrid fiber^9^, (c) 1.2 W cm^−3^–2.8 mWh cm^−3^ carbon black/graphene hybrid fiber^10^, (d) 0.79 W cm^−3^–1.73 mW cm^−3^ twisted CNT/MnO_2_ wire^2^.

Electrochemical energy storage performances of the solid-state, Ag/MnO_2_ composite sheath yarn supercapacitor comprising of two symmetric yarn electrodes coated by PVA-LiCl gel electrolyte are shown in Fig. 3. Figure 3A shows CV curve comparison (at 10 mV s^−1^) between pristine CNT and Ag/MnO_2_ composite sheath deposited CNT yarn supercapacitors. Box-like rectangular CV curve of composite sheath yarn supercapacitor without any faradic redox peaks indicates excellent capacitive characteristic. The charge storage capability of the yarn supercapacitor could be dramatically improved by present composite sheath deposition. It is notable that the contribution of the sheath coating to total charge storage performance is impressively dominant that about 50 times larger CV area is obtained. Accordingly, calculated specific areal capacitances of the single electrode are roughly proportional to the loading amount of MnO_2_ by electrochemical deposition (inset of Fig. 3D). Unless otherwise noted, capacitances are single-electrode values that are normalized with respect to the total dimension of the electrochemical active materials (presently the Ag/MnO_2_ composite sheath-CNT core yarn). The areal capacitance of pristine CNT yarn supercapacitor was about 4.9 mF cm^−2^ at 10 mV s^−1^, which is comparable to the values from previous researches (1.97–8.66 mF cm^−1^)^2,25,26^, and it dramatically increased to 322.2 mFcm^−2^ when 71.5 wt% MnO_2_ loaded for the composite sheath yarn supercapacitor. CV curves with various scan rates (at 2, 30, and 100 mV s^−1^) and galvano-static charge/discharge curves with various current densities (at 50, 100, and 200 mA cm^−3^) for composite sheath yarn supercapacitor are plotted in Fig. 3B,C, respectively. Moreover, this pseudocapacitive characteristic can also be obtained when working voltage range is extended up to 1.2 V (Fig. S5). Linear time-dependent change characteristic without any redox reaction at a constant current density in galvano-static curves also indicates the capacitive characteristic of present composite yarn supercapacitor. The areal capacitance of yarn type supercapacitors shows only moderate capacitance enhancement (18.6~512% capacitance increases^27–30^) by pseudocapacitive material loading. In contrast, the dramatic capacitance enhancement is achieved by present composite sheath yarn supercapacitor as shown in Fig. 3D. Over 50 times higher areal capacitance than pristine CNT yarn (from 4.9 to 322.2 mF cm^−2^) was achieved by incrementing the active material loading level as shown inset of Fig. 3D. As a result, composite sheath supercapacitor shows higher areal capacitance compared with previously reported plain core-shell structured yarn supercapacitors^1,4–6^.

Areal and volumetric specific capacitances are plotted in Fig. 3E. The highest values of the areal and volume normalized specific capacitances were calculated to be 322.2 mF cm^−2^ and 208.1 F cm^−3^, respectively, from CV curves at 2 mV s^−1^ scan rate. Resulting energy and power densities of the composite sheath supercapacitor are compared with previously reported other yarn supercapacitors as shown in Fig. 3F and Table S1. At 1.2 V, the calculated areal and volumetric energy densities of present composite sheath yarn supercapacitor were 18.3 μWh cm^−2^ and 11.8 mWh cm^−3^, respectively (normalized by total device volume including both yarn electrodes and PVA-LiCl gel electrolyte coating). The composite sheath yarn supercapacitor’s energy density is higher than other flexible yarn type supercapacitors^2,8–10^ as shown in Table S1. The volumetric energy density value is higher than previously reported ZnO-doped MnO_2_ Core/shell nanocables (0.04 mWh cm^−3^)^8^, poly(vinyl alcohol)/graphene hybrid fiber (5.97 mWh cm^−3^)^9^, carbon black/graphene hybrid fiber (2.8 mWh cm^−3^)^10^ and twisted CNT/MnO_2_ wire (1.73 mWh cm^−3^)^2^.

**Figure 4:**
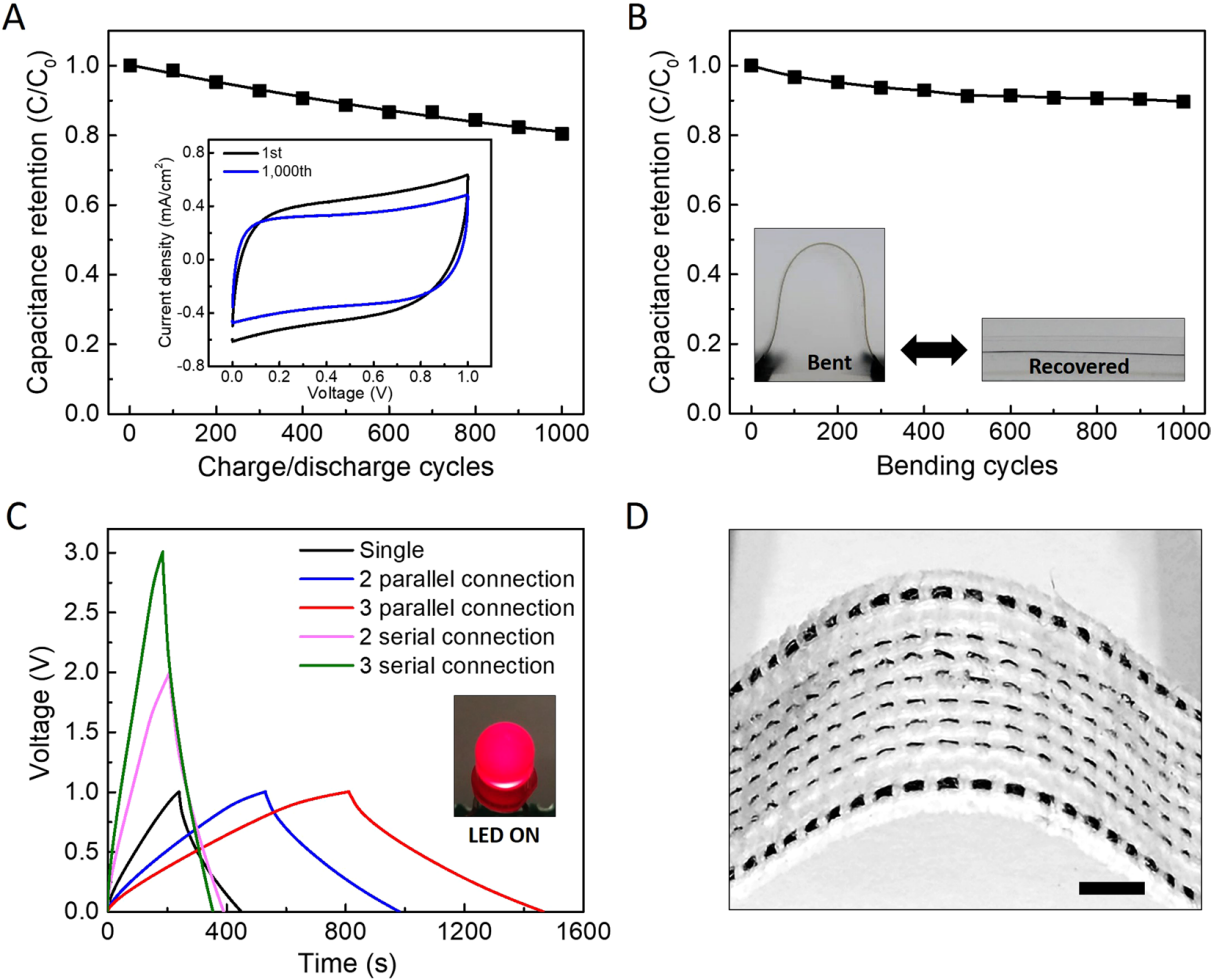
(**A**) Capacitance retention versus charge/discharge cycles. The inset compares CV curves before and after 1,000 charge/discharge cycles. (**B**) Capacitance retention depending on bending cycles. The inset shows optical images of bent and recovered Ag/MnO_2_ composite sheath electrode. (**C**) Galvano-static curves (measured at 25 mA) in parallel and serial connections. The inset shows optical image of lightened LED in 2-parallel and 3-serial connections. (**D**) The optical image of woven textile application with 6 wefts of 2-plied yarn Ag/MnO_2_ composite electrodes.

Capacitance retention performance of the solid-state, composite sheath yarn supercapacitor versus charge/discharge cycle was characterized in Fig. 4A. Area of initially measured CV curve was retained about 80% after 1000 charge/discharge cycle at 10 mV/s (inset of Fig. 4A). Despite the high loading of brittle active materials, present sheath-core supercapacitor mechanically strong and flexible. About 130 MPa yarn strength is achieved at 1.6% tensile deformation as shown in Fig. S6. To demonstrate the excellent flexibility, capacitance retention was also measured during dynamically applied bending with 170 bending degree and found to be about 90% capacitance retention after 1000 times bending test as shown in Fig. 4B. The solid-state supercapacitors can be connected in serial or parallel directions. The proportional increase in voltage and current were observed in CV curves for serial and parallel connected supercapacitors, respectively (Fig. 4C). Moreover, to demonstrate the possibility of wearable energy storage application, 2 cm long yarns were sewn into in commercial textile as shown in Fig. 4D. Due to high mechanical strength, the arrays of original yarns in the textile were successfully replaced by the embedded yarn electrodes.

## Conclusion

Herein, we achieved synergistic effect of high loading level of pseudocapacitive MnO_2_ and reasonably improved rate-capability. The interconnected networks provide electrical contact between MnO_2_ aggregates and adjacent Ag layer in the composite sheath. The conductive Ag inter layers shorten the solid-state charge diffusion length in the MnO_2_. Moreover, generated electrons during the charge/discharge process can be collected rapidly by the adjacent Ag layer, therefore, the great extents of MnO_2_ could be loaded onto the surface of CNT core fiber electrode without a significant rate-capability degradation (42.6% in comparison to pristine MnO_2_ sheath’s 21.2%). Moreover, thanks to high MnO_2_ loading level of the composite sheath-core yarn supercapacitor, excellent charge storage capabilities (specific areal and volumetric capacitances: 322.2 mF cm^−2^ and 208.1 F cm^−3^) and according energy density (area and volumetric energy densities: 15.3 μWh cm^−2^ and 8.3 mWh cm^−3^) were achieved.

## Method

### Preparation of Ag/MnO_2_ Composite Sheath Supercapacitor

Commercial CNT yarn (Muratec, Japan) is used as a current collector. One end of the 30~40 μm diameter CNT yarn electrode was connected electrically to Cu wire using silver paste and cover it with epoxy. First of all, the electrochemical deposition of MnO_2_ onto the CNT yarn electrode was performed using a potentiostatic method. About 1.3 V (vs Ag/AgCl as a reference electrode and Pt mesh as a counter electrode in a three-electrode system) was applied for 5 minutes using an electrochemical analyzer (CHI 627b system, CH Instruments, Austin, TX) in a solution containing 0.02 M MnSO_4_.5H_2_O and 0.2 M Na_2_SO_4_. After the first MnO_2_ deposition, the deposition of Ag performed onto MnO_2_-depositied CNT yarn electrode using a potentiostatic method. About −1 V (vs Ag/AgCl as a reference electrode and Pt mesh as a counter electrode in a three-electrode system) was applied for 10 seconds using an electrochemical analyzer in solution containing 0.02 M AgNO_3_ and 0.2 M Na_2_SO_4_. The total MnO_2_ and Ag alternative depositions were performed 10 and 9 times, respectively. The PVA–LiCl gel electrolyte containing 3 g PVA (Mw 146,000–186 000) and 6 g LiCl in 30 mL deionized water was prepared. The solution was heated on 160~180 °C until they dissolve in the water. Fabrication of the Ag/MnO_2_ composite sheath yearn supercapacitor was completed by coating the 10 wt% PVA–LiCl gel electrolyte on parallel, symmetric Ag/MnO_2_ composite sheath electrodes. PVA wt% is key parameter to control the viscosity of prepared gel electrolyte. For ideal case, the gel electrolyte needs to have proper viscosity so that can be easily coated on the surface of the yarn electrode when coating. At the same time, it should maintain yarn shape with enough viscosity after drying process. We experimentally find out that 10 wt% is the optimized concentration condition. And we used PVA-LiCl electrolyte because MnO_2_ is degraded because it reacts with acids and bases. The reduction reaction with the acid cause MnO_2_ to be degraded by the following reaction.

$${{\rm{MnO}}}_{2}+2{{\rm{H}}}_{2}{{\rm{SO}}}_{4}\to 2{{\rm{MnSO}}}_{4}+{{\rm{O}}}_{2}+2{{\rm{H}}}_{2}{\rm{O}}$$

$${{\rm{MnO}}}_{2}+4{\rm{HCl}}\to {{\rm{MnCl}}}_{2}+{{\rm{Cl}}}_{2}+2{{\rm{H}}}_{2}{\rm{O}}$$

The oxidation reaction with the alkali cause MnO_2_ to be degraded by the following reaction.

$$2{{\rm{MnO}}}_{2}+4{\rm{KOH}}+{{\rm{O}}}_{2}\to 2{{\rm{K}}}_{2}{{\rm{MnO}}}_{4}+2{{\rm{H}}}_{2}{\rm{O}}$$

All products used in this experiment were purchased from Sigma-Aldrich without additional chemical treatments. The weight of the CNT yarn and electrodes was measured with an electronic balance. The mass loading weight was calculate from the formula *loaded mass weight* (*sheath*) = *Total electrode weight* − *CNT yarn weight* (*core*). The loaded wt% was calculated from the formula *loaded mass weight* (*sheath*)*/Total electrode weight*.

### Characterization

All electrochemical measurements for the solid-state yarn supercapacitor used the two-electrode configuration and the electrochemical analyzer. X-ray diffraction data were measured by x-ray diffractometer (SmartLab, Rigaku). Cyclic voltammetry and chronopotentiometry measurements for all investigated supercapacitors were made using a electrochemical analyzer (CHI 627b, CH Instrument). Nyquist curves were measured using another electrochemical analyzer (Reference 600, Gamry Instrument). Scanning electron microscope images were obtained using a Zeiss Supra 40 SEM.

### Specific Capacitance Calculation

The capacitance of Ag/MnO_2_ composite sheath supercapacitor was calculated from the CV curves. From *C* = *I*/(d*V*/d*t*), where *I* is average current and dV/dt is the voltage scan rate. The specific areal capacitance for each electrode in a supercapacitor having equal anode and cathode capacitances was calculated using *C*_*A*_ = 4*C*/*A*, where *A* is the total surface area of the anode and cathode. Also, the specific volumetric capacitance for each electrode in a supercapacitor having equal anode and cathode capacitances was calculated using *C*_*v*_ = 4*C*/V, where V is the total volume of the anode and cathode. The specific energy density was calculated from the equation *E* = 1/2*CV*^2^.

### Cross-sectional Characterization

The Ag/MnO_2_ composite sheath yarn was cut and polished along its diameter using Ga ions in a Focused Ion Beam (FIB NOVA 200). Microstructural and chemical analyses were carried out at Zeiss SUPRA 40 Gemini EDAX and Zeiss-LEO Model 1530. Samples were coated by sputtering with gold for imaging purposes. ImageJ was used as a tool for image-based quantitative digital analysis of the porosity of the cross-section of the composite yarn.

## Electronic supplementary material

Supplementary Information

## References

[1] Choi C (2014). Flexible supercapacitor made of carbon nanotube yarn with internal pores. Adv. Mater..

[2] Ren J (2013). Twisting carbon nanotube fibers for both wire-shaped micro-supercapacitor and micro-battery. Adv. Mater..

[3] Yu D (2013). Controlled functionalization of carbonaceous fibers for asymmetric solid-state micro supercapacitors with high volumetric energy density. Adv. Mater..

[4] Choi C (2016). Elastomeric and dynamic MnO2/CNT core-shell structure coiled yarn supercapacitor. Adv. Energy Mater..

[5] Choi C (2015). Stretchable, weaveable coiled carbon nanotube/MnO_2_/polymer fiber solid-state supercapacitor. Sci. Rep..

[6] Zhang Z (2015). Superelastic supercapacitors with high performances during stretching. Adv. Mater..

[7] Lee JA (2013). Ultrafast charge and discharge biscrolled yarn supercapacitors for textiles and microdevices. Nat. Commun..

[8] Yang P (2013). Hydrogenated Zno Core-shell nanocables for flexible supercapacitors and self-powered systems. ACS Nano.

[9] Chen S (2016). Conductive, tough, hydrophilic poly(vinyl alcohol)/graphene hybrid fibers for wearable supercapcitors. J. Power Sources.

[10] Ma W (2016). Hierarchically porous carbon black/graphene hybrid fibers for high performance flexible supercapacitors. RSC Adv..

[11] Xie K, Wei B (2011). Materials and structures for stretchable energy storage and conversion devices. Adv. Mater..

[12] Xie K (2011). Polyaniline nanowire array encapsulated in titania nanotubes as a superior electrode for supercapaciotrs. Nanoscale..

[13] Xie K (2011). Highly ordered iron oxide nanotube arrays as electrodes for electrochemical energy storage. Electrochem. Commun..

[14] Wei W, Cui X, Chen W, Lvey DG (2011). Manganese oxide-based materials as electrochemical supercapacitor electrodes. Chem. Soc. Rev..

[15] He Y (2013). Freestanding three-dimensional graphene/MnO_2_ composite networks as ultralight and flexible supercapacitor electrodes. ACS NANO.

[16] Xu C, Kang F, Li B, Du H (2013). Recent progress on manganese dioxide based supercapacitors. J. Mater. Res..

[17] Gogotsi Y, Simson P (2011). True performance metrics in electrochemical energy storage. Science.

[18] Zhang M, Atkinson KR, Baughman RH (2004). Multifunctional carbon nanotube yarns by downsizing an ancient technology. Science.

[19] Dong X (2012). Synthesis of a MnO_2_-graphene foam hybrid with controlled MnO_2_ particle shape and its use as a supercapacitor electrode. Carbon.

[20] Ren J, Bai W, Guan G, Zhang Y, Peng H (2013). Flexible and weaveable capacitor wire based on a carbon nanocomposite fiber. Adv. Mater..

[21] Choi C (2016). Improvement of system capacitance via weavable superelastic biscrolled yarn supercapacitors. Nat. Commun..

[22] Choi, C. *et al*. Weavable asymmetric carbon nanotube yarn supercapacitor for electronic textiles. *RSC Adv*. **8** 13112 (13120). 10.1039/c8ra01384e PMC9079689 35542516

[23] Chen X (2013). Peng, Novel electric double-layer capacitor with a coaxial fiber structure. Adv. Mater..

[24] Yu D (2014). Controlled functionalization of carbonaceous fibers for asymmetric solid-state micro supercapacitors with high volumetric energy density. Adv. Mater..

[25] Bae J (2011). Fiber supercapacitors made of nanowire-fiber hybrid structures for wearable/flexible energy storage. Angew. Chem. Int. Ed..

[26] Meng Y (2013). All-graphene core-sheath microfibers for all-solid-state, stretchable fibriform supercapacitors and wearable electronic textiles. Adv. Mater..

[27] Wang K (2013). High-performance two-ply yarn supercapacitors based on carbon nanotubes and polyaniline nanowire arrays. Adv. Mater..

[28] Meng Q (2014). High-performance all-carbon yarn micro-supercapacitor for an integrated energy system. Adv. Mater..

[29] Kou L (2014). Coaxial wet-spun yarn supercapacitors for high-energy density and safe wearable electronics. Nat. Commun..

[30] Ma Y (2015). Conductive graphene fibers for wire-shaped supercapacitors strengthened by unfunctionalized few-walled carbon nanotubes. ACS Nano.

