# Supplementary material for: Ag/MnO2 Composite Sheath-Core Structured Yarn Supercapacitors
Source: Sci Rep. 2018 Sep 6;8:13309. doi: 10.1038/s41598-018-31611-2 (PMC6127153; doi:10.1038/s41598-018-31611-2)
Supplement: Supplementary file 1 — Supplementary Information [file 41598_2018_31611_MOESM1_ESM.doc]

# Supplementary Information

# Ag/MnO_2_ Composite Sheath-Core Structured Yarn Supercapacitors

**Ji Hwan Kim^1,+^, Changsoon Choi^1,2^,**^+^**, Jae Myeong Lee^1^, Mônica Jung de Andrade^3^, Ray H. Baughman^3^ and Seon Jeong Kim^1^,***

^1^ *Center for Self-powered Actuation, Department of Biomedical Engineering, Hanyang University, Seoul 04763, Korea*

^2^ *Division of Smart Textile Convergence Research, Daegu Gyeongbuk Institute of Science and Technology (DGIST), Daegu 42988, Korea*

^3^ *The Alan G. MacDiarmid NanoTech Institute, University of Texas at Dallas, Richardson, TX 75083, USA*

* To whom correspondence should be addressed. E-mail: [sjk@hanyang.ac.kr](mailto:sjk@hanyang.ac.kr)

^+^ These authors are equally contributed to this work.

***
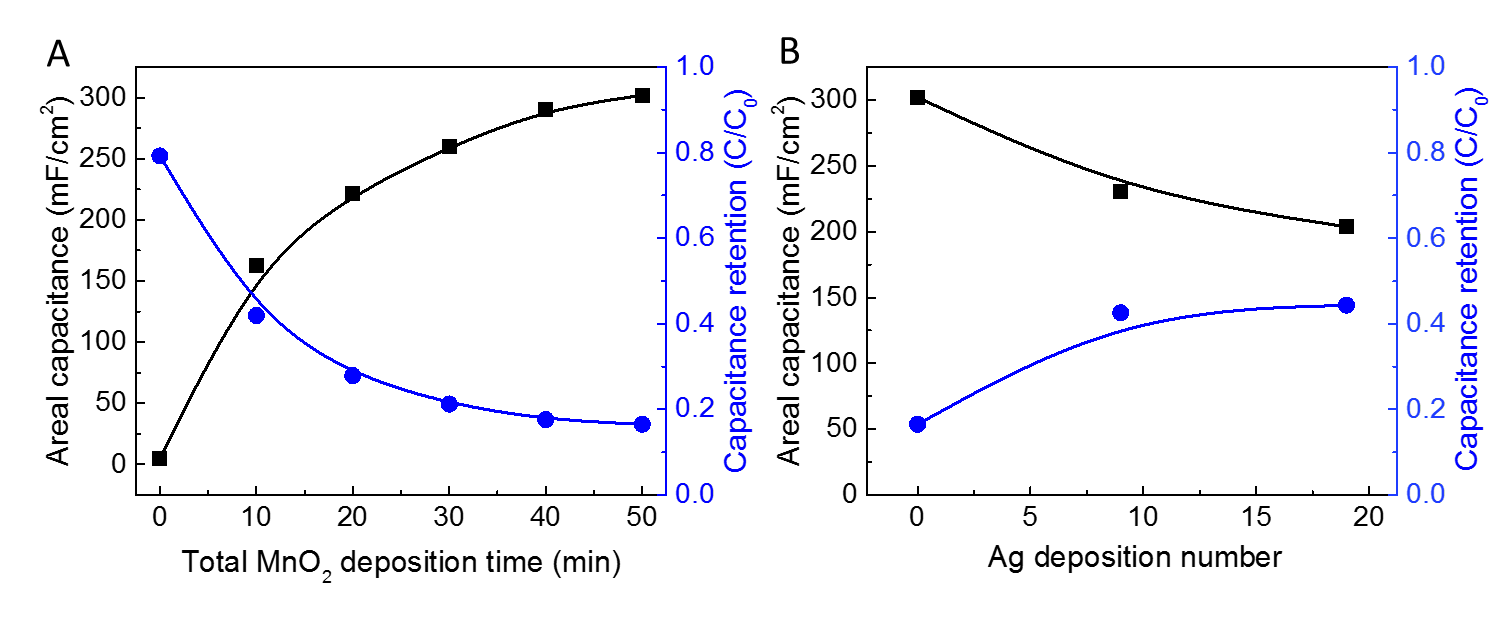
***

**Figure S1**. (A) Areal capacitance and its retention (at 10mV s^-1^) depending on total MnO_2_ deposition time. (B) Areal capacitance and its retention depending on Ag deposition number. Each Ag deposition was performed for 10 seconds at -1 V.

**
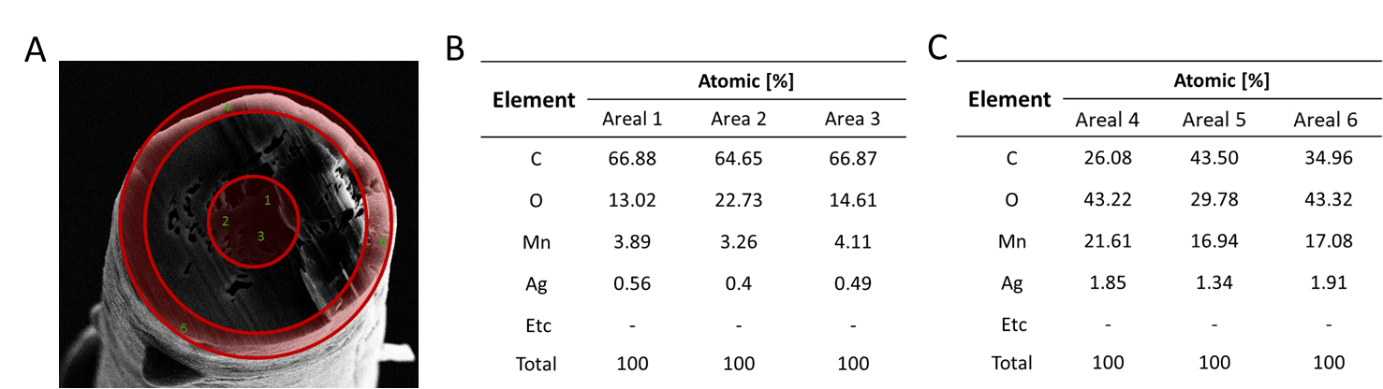
**

**Figure S2**. (A) The cross-sectional SEM image of Ag/MnO_2_ composite sheath electrode. (B), (C) Graphs shows the atomic % of core (Area 1, 2, and 3) and composite sheath (Area 4, 5, and 6), respectively.

**
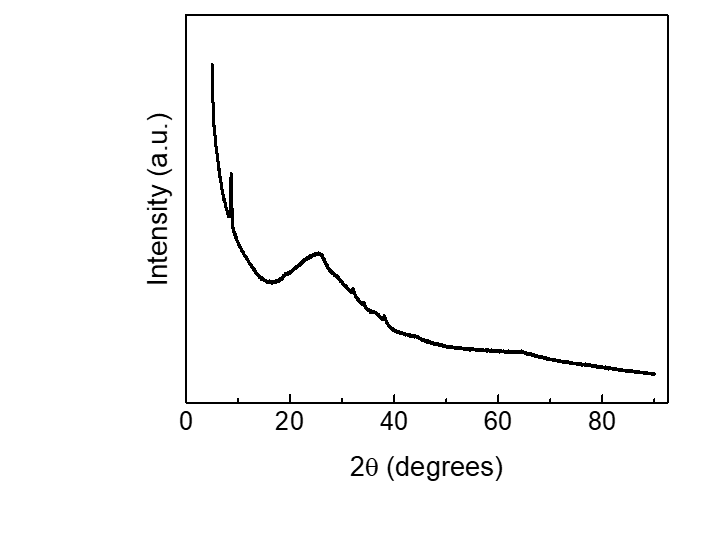
**

**Figure S3.** XRD pattern of Ag/MnO_2_ composite sheath electrode.

**
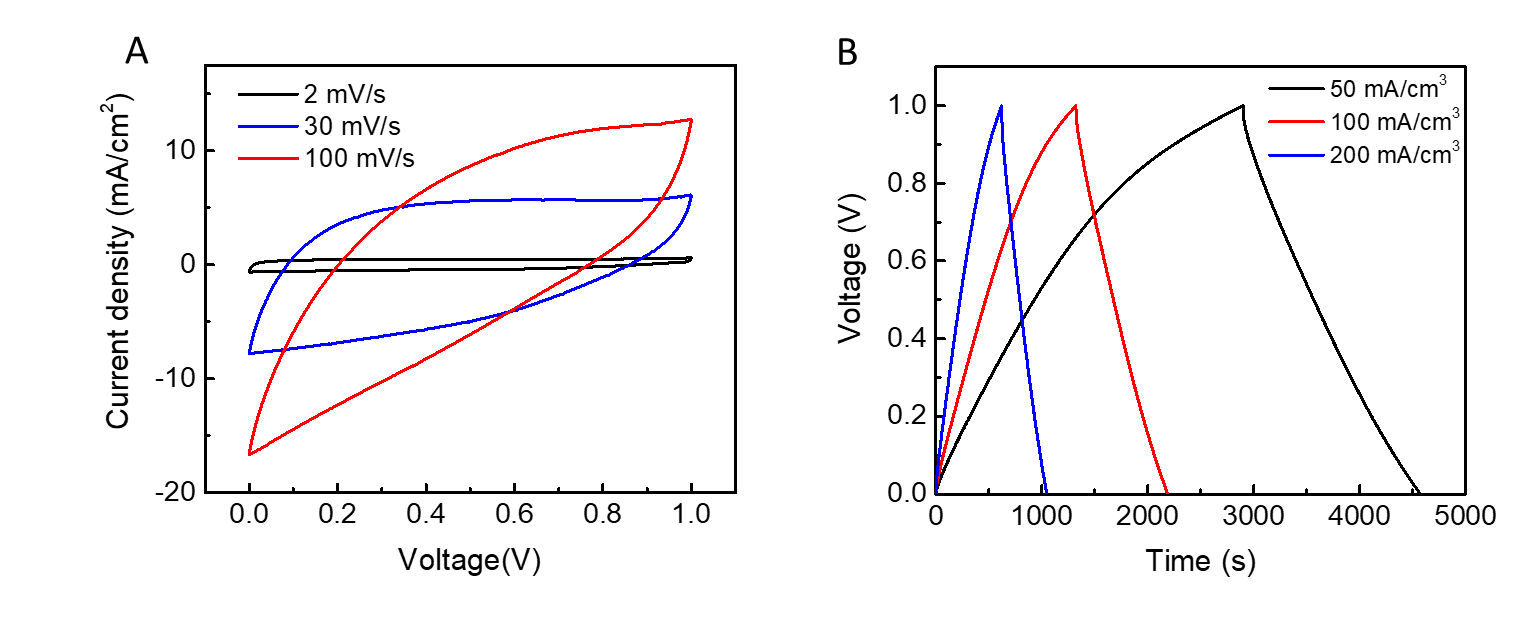
**

**Figure S4**. (A) CV (measured from 10 to 100 mV s^-1^) and (B) Galvano-static curves (measured from 50 to 200 mA cm^-3^) of pristine MnO_2_ sheath supercapacitor. The supercapacitors comprise two symmetric electrodes coated by PVA/LiCl gel electrolyte.

**
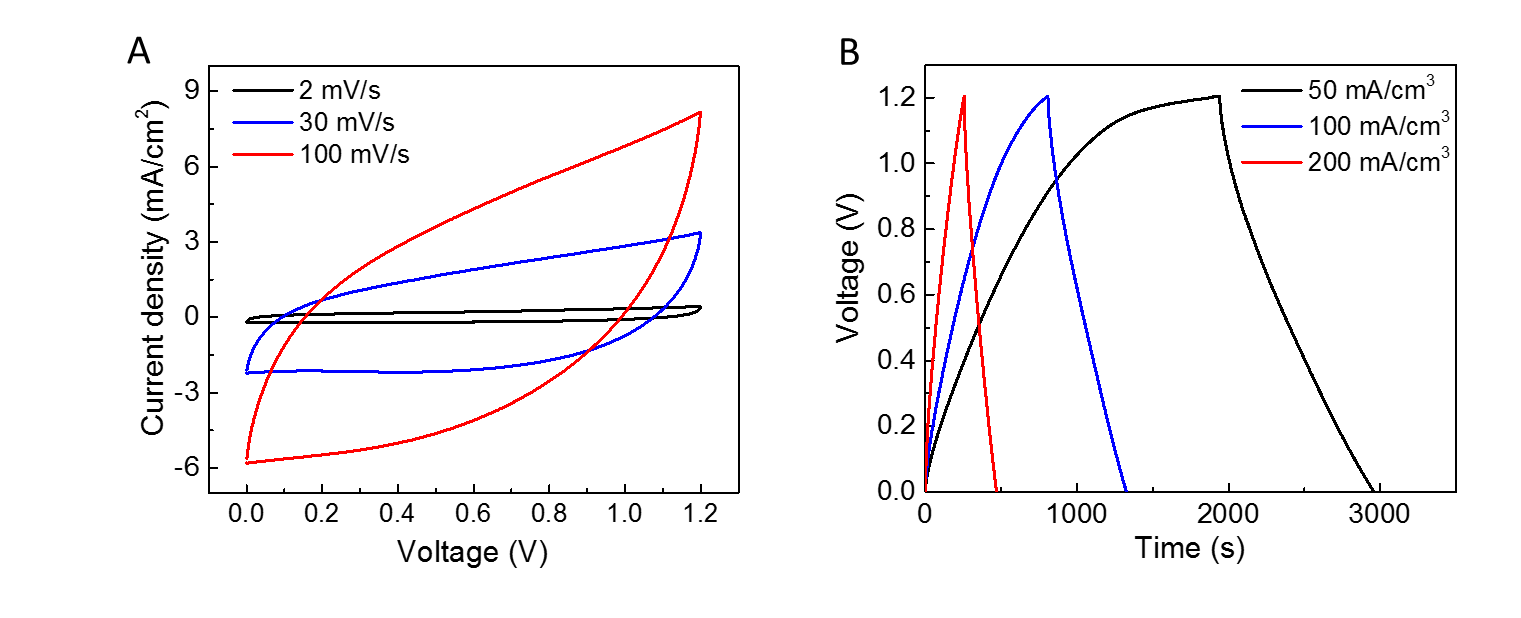
**

**Figure S5**. (A) CV (measured from 2 to 100 mV s^-1^) and (B) Galvano-static curves (measured from 50 to 200 mA cm^-3^) of Ag/MnO_2_ composite sheath supercapacitor at 1.2 V. The supercapacitors comprise two symmetric electrodes coated by PVA/LiCl gel electrolyte.

**Table S1.** Areal and volumetric capacitances and energy densities comparison table.

| **Fiber or yarn electrode** | **C_A_**  **[mF cm^-2^]** | **C_V_**  **[F cm^-3^]** | **E_A_**  **[µWh cm^-2^]** | **E_V_**  **[mWh cm^-3^]** |
| --- | --- | --- | --- | --- |
| **Ag/MnO_2_ Composite sheath** | **322.3** | **208.1** | **15.3-18.3** | **8.3-11.8** |
| CNT/Active carbon^[28]^ | 148.4 | 194 | 3.29 | 3.7 |
| RGO/CNT spinning^[29]^ | 177 | 158 | 3.84 | 3.5 |
| RGO/CNT spinning^[30]^ | - | 155.2 | - | 3.4 |
| CNT/MnO_2_^[1]^ | - | 100.2 | - | 3.5 |
| CNT/MnO_2_^[2]^ | 3.57 | - | - | 1.73 |
| CNT/PEDOT^[7]^ | 73 | 179 | - | 1.4 |
| MnO_2_/ZnO^[8]^ | 138.7 | - | - | 0.04 |
|  |  |  |  |  |


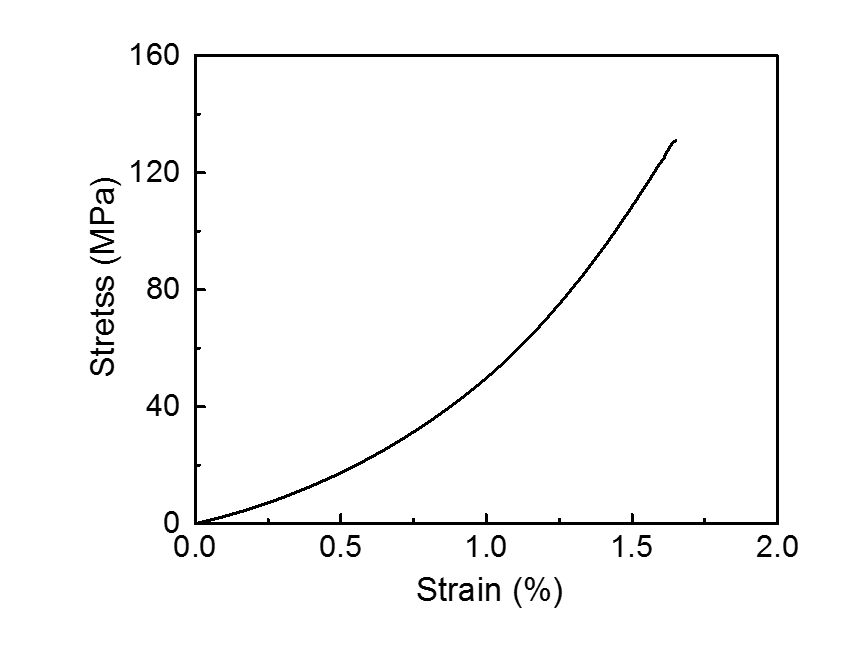


**Figure S6.** SS curve of Ag/MnO_2_ composite yarn electrode.
